# 

**Published:** 1893-10-28

**Authors:** 


### CONTENTS.

- **Death Certification and Murder** - 49

- **Around the Hospitals** - 50

- **Modern Aspects of Pain in Medicine** - 51

- **The History and Capabilities of Herbal Simples.** By W. T. Fernie, M.D.—Good King Henry - 52

- **The Medical Work of the Local Government Board.**—The Manure Conundrum - 53

- **The Field of Science** - 54

- **Annotations.**—Sir Andrew Clark's Illness—Cruelty in Irish Parents—Object Lessons from Lanarkshire - 55

- **Notes and News** - 56

- **Scraps and Gleanings** - 64

- **The Book World of Medicine and Science** - vii

- **The Hospital Clinic—**

  - St. Mary's Hospital, London.—On the Treatment of Otorrhœa in the Aural Department. III. - 57

  - Liverpool Royal Infirmary.—The Treatment of Empyema - 58

  - The Treatment of Summer Diarrhœa of Children. - 52

  - Hospitals in India.—I. The Bengal Presidency - 60

- **The Institutional Workshop—**

  - Within the Hospitals.—The Poplar Hospital - 61

  - Practical Departments.—Gas Stoves - 62

  - The Story of the Insane from Year to Year - 62

  - Middlesex Hospital.—Many Internal Improvements - 62

  - Hospital Worthies.—Mr. Henry Dobbin - 63

- **Medical Vacancies and Appointments** - x

### EXTRA SUPPLEMENT.—THE NURSING MIRROR.

- **News from the Nursing World.**—Our Christmas Competitions—Can Nurses be Cowards?—Invalid Children—Amateur or Professional?—A New Children's Ward—Novel Methods—Strictly Confidential—To Work, not to Learn—Discharged Cured—A New Nurses' Home—Short Items - xxxi
- **On the Nursing of Diseases of the Nervous System.**—IV. Meningitis—Paraplegia - xxxiii
- **Nursing in South Africa,**—The Lepers at Robben Island - xxxiv
- **Thoughts on District Nursing** - xxxiv
- **Hospitals in Switzerland.**—Lucerne - xxxv
- **Minor Appointments** - xxxv
- **Libraries for Nurses** - xxxv
- **United States of America.**—Notes from New York City—The Floating Hospital. Notes from Philadelphia - xxxvi
- **Nursing at Kimberley** - xxvvii
- **Novelties for Nurses** - xxxvii
- **For Reading to the Sick.**—Misunderstood - xxxvii
- **Lectures to Ladies** - xxxviii
- **Notes and Queries** - xxxviii
- **Appointments** - xxxviii
- **The Book World for Women and Nurses** - xxxix

